# The association between major complications of immobility during hospitalization and quality of life among bedridden patients: A 3 month prospective multi-center study

**DOI:** 10.1371/journal.pone.0205729

**Published:** 2018-10-12

**Authors:** Xinjuan Wu, Zhen Li, Jing Cao, Jing Jiao, Yingli Wang, Ge Liu, Ying Liu, Fangfang Li, Baoyun Song, Jingfen Jin, Yilan Liu, Xianxiu Wen, Shouzhen Cheng, Xia Wan

**Affiliations:** 1 Department of Nursing, Peking Union Medical College Hospital, Chinese Academy of Medical Sciences & Peking Union Medical College, Beijing, China; 2 Operation center, Peking Union Medical College Hospital, Chinese Academy of Medical Sciences & Peking Union Medical College, Beijing, China; 3 Department of Nursing, Henan Provincial People’s Hospital, Zhengzhou, China; 4 Department of Nursing, The Second Affiliated Hospital Zhejiang University School of Medicine, Hangzhou, China; 5 Department of Nursing, Union Hospital of Tongji Medical College, Huazhong University of Science and Technology, Wuhan, China; 6 Department of Nursing, Sichuan Provincial People's Hospital, Chengdu, China; 7 Department of Nursing, The First Affiliated Hospital, Sun Yat-sen University, Guangzhou, China; 8 Institute of Basic Medical Sciences, Chinese Academy of Medical Sciences and School of Basic Medicine, Peking Union Medical College, Beijing, China; Rabin Medical Center, Beilinson Hospital, ISRAEL

## Abstract

**Purpose:**

To describe the association between major complications of immobility (pressure ulcer, pneumonia, deep vein thrombosis and urinary tract infection) during hospitalization and the patients’ health-related quality of life after discharge.

**Methods:**

The data were obtained from a multi-center study conducted in 2015. Complications of immobility during hospitalization was measured by case report form and quality of life after discharge was measured using the EQ-5D scale by telephone interview. Multilevel mixed-effects models were used to explore the association of complications and responses in the EQ-5D dimensions after controlling for important covariates.

**Results:**

Among the 20,515 bedridden patients, 2,601(12.72%) patients experienced at least one of the major complications of immobility during hospitalization, including pressure ulcer (527, 2.57%), deep vein thrombosis (343, 1.67%), pneumonia (1647, 8.16%), and urinary tract infection (265, 1.29%). Patients with any of the four complications during hospitalization reported more problems in all EQ-5D dimensions except for *pain/discomfort*, and had lower mean EQ-VAS scores than those without any complications. The four complications all showed significant associations with the proportion of reported problems in certain dimensions after adjustment for confounding variables.

**Conclusions:**

Major complications of immobility were significantly associated with reduced health related quality of life. Prevention of complications is critical to reduce the burden of decreased quality of life for bedridden patients.

## Introduction

Immobility is independently associated with the development of a series of complications, including pressure ulcer [1], deep vein thrombosis (DVT) [2], pneumonia [3], and urinary tract infection (UTI) [4]. Many studies have shown that complications of immobility could result in numerous deleterious consequences, including increased morbidity and mortality [5, 6], prolonged length of stay[7], increased hospital cost[8, 9] and contribution to global disease burden [10, 11].The impact of complications of immobility on patients’ overall well-being and functioning is a topic of growing interest in clinical research and practice [12–15]. Among these studies, health-related quality of life (HRQOL) assessments have been used as an outcome measure with hospitalized patients for many years. Assessment of HRQOL can yield information on important patient outcomes that are not obtained by routine clinical observations[16]. In addition, HRQOL defines health outcomes in broader terms than morbidity or mortality. Since it is measured from patients’ perspectives, it allows evaluation of the impact of the condition on patients’ health and daily functioning. Thus, HRQOL has become an important indicator to inform patient management and policy development and plays a valuable role in clinical decision making[17].

Understanding how complications affect long-term patient-reported outcomes (PROs) following immobility is important for health economic evaluation. Researchers have underlined the impact of a variety of complications of immobility on quality of life. A number of studies have reported the relationship between complications of immobility and significant reductions in HRQOL. Each individual complication of immobility has been associated with a significant deterioration in HRQOL [14, 18] [19] [20]. However, some deficiencies exist in current studies. Most studies have reported short-term outcomes [13, 21]. The medium and long term consequences have been less well documented. In addition, the sample size may not be sufficient to produce consistent findings. Ghanima and colleagues’ studies on DVT showed that patients who sustained DVT seem in general to report similar HRQOL compared to the general population [22], while another study showed long‑term HRQOL was significantly impaired in DVT patients compared with buddy controls and population norms[13]. It remains unclear whether these different complications have the same or different associations with HRQOL. Furthermore, most of previous studies were conducted in western countries, whereas the social and cultural settings of Chinese patients are quite different from patients in western countries. More data from Chinese population are in great demand to provide detailed information for care and management of bedridden patients.

This study was derived from a national research project, which was aimed at constructing a standardized nursing intervention model for major complications of immobility (MCI) among bedridden patients. As part of baseline investigation from the broad project, the aims of this study were to (i) investigate HRQOL of bedridden patients in the hospital setting three months after being enrolled in the study; and (ii) evaluate the association between MCI and HRQOL of bedridden patients three months after being enrolled in the study. Researchers hypothesized that patients with MCI during hospitalization would have an impaired HRQOL in comparison with HRQOL in patients who did not experience any MCI.

## Methods

### Study design

The surveys were carried out in 25 hospitals (six tertiary, 12 non-tertiary and seven community hospitals) from November 2015 to June 2016. These hospitals were located in the North, South, East, West and Central China. Patients were recruited from the following units: neuro-medical, neuro-surgical, general surgical, general medical, orthopedics, geriatric and critical care (intensive care, coronary care and respiratory care).

### Study population

**Fig 1:**
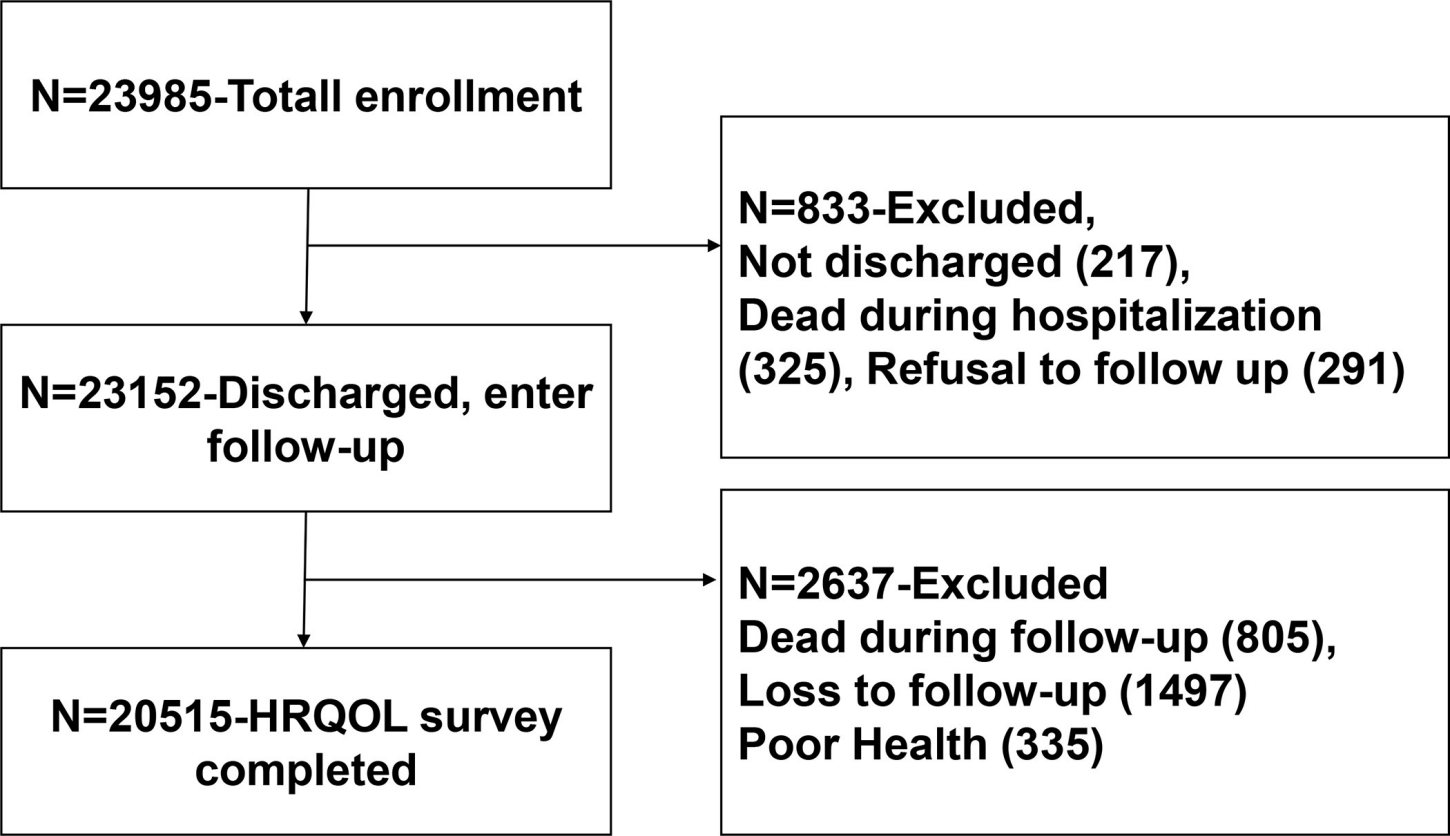
Flow chart of patient sample.

Patients were eligible for the study if they (a) were 18 years or older; (b) were bedridden for at least one day after admission in their respective wards (bedridden is defined as all of the patients’ basic physiological needs were carried out in bed except for active or passive bedside standing/wheelchair use for examination or treatment); and (c) understood the aims of the study and signed the consent form. Patients were excluded if they had more than one type of MCI at time of enrollment. For example, patients with only pneumonia on admission were included, whereas those with pneumonia and pressure ulcers were excluded. In total, 23,985 patients were initially recruited. Because this study focused on health-related quality of life, the analysis actually involved 20,515 patients who had completed the quality of life questionnaire. The flowchart of 20,515 patients’ sample is shown in Fig 1.

### Measures

#### Major complications of immobility

The MCI were measured by the case report form (CRF), which was developed by the research team, to collect demographic (age, gender) and medical information (MCI, underlying disease, length of stay, the length of bedridden experience). MCI includes pressure ulcer, DVT, pneumonia and UTI. Patients with complications that did not belong to any of these categories were classified as having zero complications for the purpose of this analysis.

Pressure ulcer was assessed by trained nurses using the National and European National Pressure Ulcer Advisory Panels Pressure Ulcer Classification System[23]. DVT, pneumonia and UTI were assessed according to medical records.

#### Quality of life

Patient-reported quality of life was measured by EQ-5D. EQ-5D is an instrument that evaluates the generic quality of life developed by EuroQol Group and widely used[24]. The EQ-5D questionnaire consists of two components, health state description and evaluation. In the descriptive part, health status is measured in terms of five dimensions (5D); *mobility*, *self-care*, *usual activities*, *pain/discomfort*, and *anxiety/depression*. In the current study, the EQ-5D-3L was used, in which each dimension is represented by one question with three severity levels (having no problems, having some or moderate problems, being unable to do/having extreme problems). The respondents were asked to choose one of the statements which best described their health on the days the survey was completed. In the evaluation part, respondents evaluated their overall health status using the visual analogue scale (EQ-VAS) with a range from 0 (the worst possible health status) to 100 (the best possible health status). The scale has been validated in Chinese populations [25, 26].

#### Other covariates

Patients’ sociodemographic characteristics (age, gender, and educational status) were self-reported by the patients, and information on medical comorbidities and hospitalizations was obtained from audits of the patients’ hospital charts and electronic health records. The age-adjusted Charlson comorbidity index was calculated based on diagnosis at discharge[27]. The modified Braden scale was also applied to measure patients’ risk at admission of adverse outcomes caused by immobilization [28].

### Data collection

Pre-trained investigators recorded the hospitalization information in the selected wards. At least two registered nurses in each participating hospital were appointed to perform patient data collection. They recorded participating patients’ information daily on a web-based electronic data capture (EDC) system. From the day of recruitment, all patients were observed and data were recorded for a period of 90 days, unless there was death or withdrawal from medical treatment. After the enrolled patients had been discharged, they received telephone follow-up every two weeks until reaching their 90^th^ day in the study. Their HRQOL was measured on the 90^th^ day. Investigators then entered the data into the EDC system.

Multiple approaches were applied to ensure accurate and reliable patient data. In the development of the CRF, items with ambiguous meanings were avoided. A pilot study was also conducted among 626 patients from all of the centers prior to the initiation of the study to ensure the comprehensibility of the CRF and the applicability of the EDC system. In the investigation training phase, all of the research assistants from the different hospitals received unified training courses on how to complete the CRF. After the training, a test was organized to measure these research assistants’ understanding of the survey content and their ability to implement the survey. Only those who passed the test were allowed to participate in the investigation. In the data collection phase, each hospital appointed a coordinator, who was responsible for internal logistics. The head nurse in each ward served as a supervisor and regularly audited data recorded by the research assistants. In addition, an independent quality control committee was in charge of supervising the data collection at the different centers. They organized a series of quality control measures to ensure the integrity and accuracy of patient information. During the hospitalization survey, 10% of hospitalized participants were randomly selected by the quality control committee. The information on these selected participants in the EDC were extracted and compared with the corresponding medical records. During the post-discharge follow-up survey, the committee randomly selected 10% of the patients under follow-up and made telephone calls to them to check the accuracy of the information collected by the research assistants. Missing or inaccurate data were gathered or corrected through additional consulting of medical records and follow-up phone calls.

#### Ethical considerations

This study was approved by the Ethical Committee of Peking Union Medical College Hospital. Written informed consent was obtained from all individual participants included in the study. If patients were unable to give written consent, their contact person gave the consent. All data were kept confidential and processed anonymously.

### Data analysis

Continuous variables were reported as means (standard deviations) and categorical variables as frequencies (percentages). Percentage of respondents reporting problems in each EQ-5D dimension and EQ-VAS score (mean) were calculated for patients with any one of MCI and patients without MCI.

The variables were organized into three levels: hospital, ward, and patient. Intra-class coefficients (ICC) were calculated to gauge the potential effect of clustering on the results. To identify adjusted effect of MCI on the five health dimensions and EQ-VAS, multivariate analysis was conducted using a multilevel mixed-effects model with two random intercept modeling the effect of the hospital and ward. The model takes into account the fact that patients from the same hospital or ward may have unmeasured characteristics that make their responses to the questionnaire within the hospital or ward more alike than across different hospital or ward. The mixed-effects model analyses were run separately for each response variable (e.g., *mobility*, *self-care*, *usual activities*, *pain/discomfort*, *anxiety/depression* and EQ-VAS.) to evaluate the effect of MIC. Rated levels of EQ-5D dimension were coded as a number 0 or 1, with 0 indicating an absence of problems, and 1 indicating some problems or extreme problems. EQ-VAS was treated as continuous variable. Covariates that were considered to be important impact factors in the quality of life based on prior literature and univariate analysis were taken as candidates for inclusion in the models. Hospital-level variables included hospital type (tertiary and non-tertiary hospitals), ward-level variables included ward classification (medicine, surgery and critical care). Patient-level variables including age, gender, education, length of bedridden experience, length of stay, mobility at admission, modified Braden score, number of diagnoses when discharged, age adjusted Charlson comorbidity index were treated as control variables, and were forced into the model regardless of their statistical significance. The assumptions of normality, linearity, homoscedasticity, and absence of multicollinearity were checked before building the mixed-effects model for EQ-VAS. The statistical analyses were carried out using SAS 9.4 for Windows (SAS Institute, Cary, North Carolina, USA). All tests were 2-tailed with p < 0.05 considered significant.

## Results

### Characteristics of respondents

A total of 20,515(85.53%) patients provided complete HRQOL data and were included in the final data set. Table 1 displays characteristics of patients with MCI and patients without MCI. 11,513 (56.12%) patients were male. Participants’ age ranged from 18 to 109 years, with an overall mean age of 55.57 years. Among all participants, 45.79% of patients had junior or senior high school education, and 15.62% of patients had a college degree or above. 16,434 (80.11%) patients had health insurance. 11649(56.78%) were admitted to hospital by walking, 12732 (62.84%) scored more than 19 on modified Braden scale at admission 7,176 (34.98%) patients were bedridden between 1 to 3 days during hospitalization. Regarding length of stay, 8,738 (42.59%) patients stayed more than 15 days, and 8,090 (39.43%) patients stayed 8 to 14 days. 9,470 (48.24%) patients had at least 2 documented medical diagnoses when discharged. The mean score of age adjusted Charlson comorbidity index was 3.07(2.28), while 6972(33.98%) scored 3 or 4. Compared with those who did not experience any MCI during hospitalization, patients with MCI tended to be older, less educated, with less health insurance, poor mobility as well as a lower Modified Braden score at admission. They were bedridden longer and had greater length of stay. They also had more comorbidity and a higher score of age adjusted Charlson comorbidity index.

**Table 1:** Baseline characteristics of patients with MCI and patients without MCI (n = 20515).

Variables	Total group	Experiencing MCI during hospitalization		t/χ^2^	p value
		at least 1 type of MCI	none		
**No. of patients**	20515	2610	17905		
**Gender**					
male	11513(56.12)	1504 (57.62)	10009(55.90)	2.749	0.0973
female	9002(43.88)	1106(42.38)	7896(44.10)		
**Age in years**	55.57±16.46	61.43±16.82	54.71±16.23	19.15	<0.001
**Age group**					
18~34	2556(12.46)	210(8.05)	2346(13.10)	449.00	<0.001
35~54	6857(33.42)	636(24.37)	6221(34.74)		
55~74	8422(41.05)	1111(42.57)	7311(40.83)		
≥75	2680(13.06)	653(25.02)	2027(11.32)		
**Highest education level**					
Primary school or below	7923(38.62)	1226(46.97)	6697(37.40)	97.161	<0.001
Junior or senior high school	9387(45.76)	1081(41.42)	8306(46.39)		
College degree or above	3205(15.62)	303(11.61)	2902(16.21)		
**Health insurance**					
No	4081(19.89)	596(22.84)	3485(19.46)	16.248	<0.001
Yes	16434(80.11)	2014(77.16)	14420(80.54)		
Mobility at admission					
Walking	11649(56.78)	672(25.75)	10977(61.31)	1258.74	<0.001
Using a wheelchair	2165(10.55)	349(13.37)	1816(10.14)		
Carried on a stretcher	6701(32.66)	1589(60.88)	5112(28.55)		
Modified Braden score at admission					
≤19	7528(37.16)	1489(57.20)	6039(34.20)	514.00	<0.001
>19	12732(62.84)	1114(42.80)	11618(65.80)		
**Length of bedridden days**					
1~3	7176(34.98)	225(8.62)	6951(38.82)	18888.05	<0.001
4~7	6279(30.61)	523(20.04)	5756(32.15)		
≥8	7060(34.41)	1862(71.34)	5198(29.03)		
**Length of hospital stays in days**					
1~7	3687(17.97)	264(10.11)	3423(19.12)	371.36	<0.001
8~14	8090(39.43)	789(30.23)	7301(40.78)		
≥15	8738(42.59)	1557(59.66)	7181(40.11)		
**Number of diagnosis when discharge (n = 19630)**					
1~2	9470(48.24)	688(27.34)	8782(51.31)	749.04	<0.001
3~4	5015(25.55)	639(25.40)	4376(25.57)		
≥5	5145(26.21)	1189(47.26)	3956(23.12)		
**Comorbidities(n = 19630)**					
ACCI, mean(SD)	3.07(2.28)	3.80(2.24)	2.96(2.27)	17.684	<0.001
ACCI group					
None(ACCI 0)	2724(13.28)	179(6.86)	2545(14.21)	364.01	<0.001
Mild(ACCI 1–2)	6158(30.02)	560(21.46)	5598(31.27)		
Moderate(ACCI 3–4)	6972(33.98)	964(36.93)	6008(33.55)		
Severe(ACCI≥5)	4661(22.72)	907(34.75)	3754(20.97)		

Note: MICs: major immobility complications ACCI: Age adjusted Charlson comorbidity index.

### MCI during hospitalization

Of 20,515 patients, 2,610 experienced at least one type of MCI. Of these 2,610 patients, the majority of patients (2,424, 92.87%) experienced 1 complication, 173 (6.63%) patients experienced 2 complications and the remaining 13 patients (0.50%) experienced 3 complications. The most common complication was pneumonia (1,647, 8.16%), followed by pressure ulcer (527, 2.57%), DVT (343, 1.67%), and UTI (265, 1.29%). See Table 2.

**Table 2:** Type of MCI in 20515 patients.

Complication	No. of Patients	%
Pressure ulcer	527	2.57
Deep vein Thrombosis	343	1.67
Pneumonia	1647	8.16
Urinary Tract Infections	265	1.29
Total*	2610	12.72

Note: MICs: major immobility complications

*A patient could have had more than one complication.

### HRQOL assessment based on presence and type of MCI

**Fig 2:**
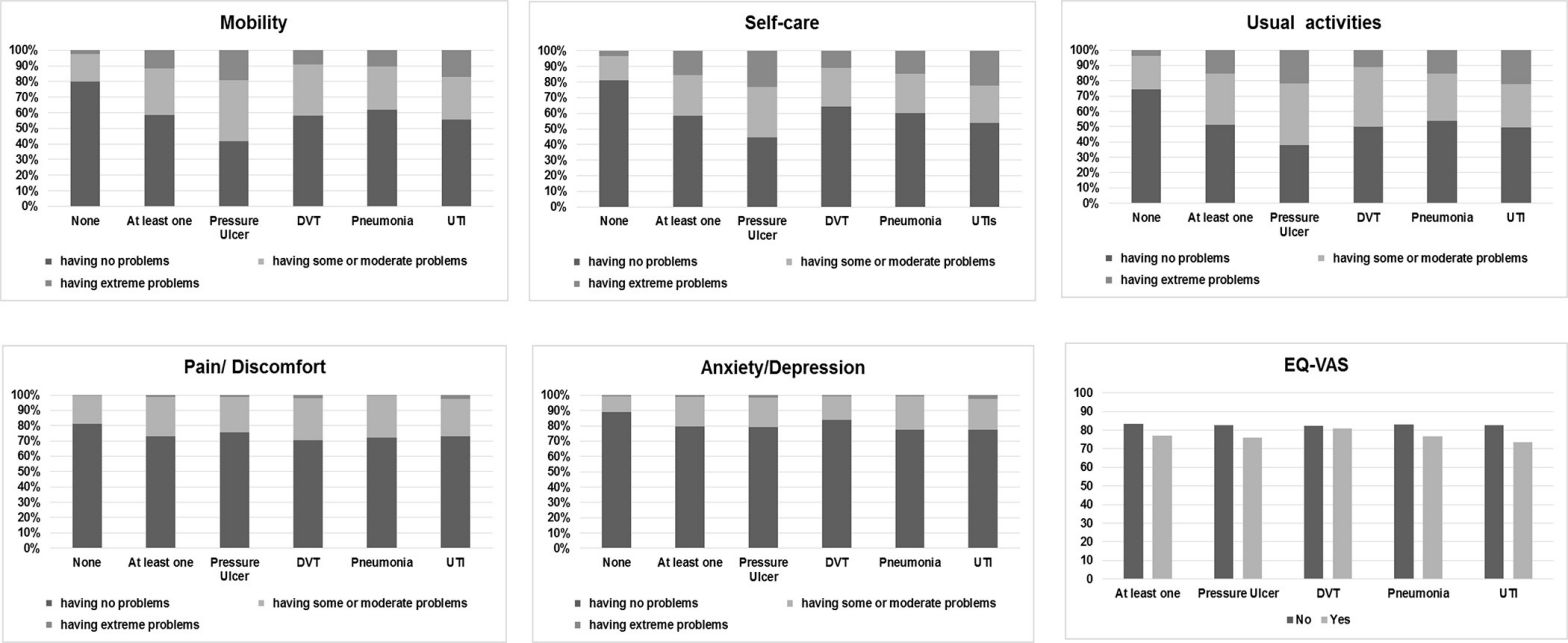
The distribution of the proportion of problems reported by patients with MCI in relation to five domains of the EQ-5D and EQ-VAS scores.

Fig 2 displays the distribution of the proportion of reported problems in EQ-5D dimensions from each of the 6 groups of patients (patients without MCI, patients with at least 1 type of MCI, patients with pressure ulcer, patients with DVT, patients with pneumonia, and patients with UTI).

20.13% of patients without MCI had problems with *mobility*, 19.11%with *self-care*, 25.8% with *usual activities*, 18.82% with *pain/discomfort* and 10.9% with *anxiety/depression*. The mean EQ-VAS score was 83.23 for non-MCI cases. In comparison with patients without MCI, 41.53% of patients with at least 1 type of MCI had problems with *mobility*, 41.45% with *self-care*, 48.7% with *usual activities*, 27.09% with *pain/discomfort* and 20.5% with *anxiety/depression*. The mean score of EQ-VAS was77.02 for the group of patients with at least 1 type of MCI.

Examining HRQOL of the remaining 4 groups, problems with *usual activities* were most reported among the five dimensions. (pressure ulcer 61.86%, DVT 50.15%, pneumonia 46.23% and UTI 50.56%). *Mobility* was the next frequent problem reported by the pressure ulcer group (58.26%) and the DVT group (41.98%), but *Self-care* was the next frequent problem reported by the pneumonia group (39.79%) and the UTI group (46.03%). the mean score of EQ-VAS for these 4 groups of patients ranged from 73.55 to 80.92.

### The association between MCI and HRQOL

Table 3 shows the result of multilevel mixed-effects model analysis after adjustment of a series of covariates. Patients experiencing at least 1 type of MCI were significantly more likely to report problems with *mobility*(*β* = 0.229,95%CI:0.127,0.331), *self-care*(*β* = 0.250,95%CI:0.146,0.354), *usual activities*(*β* = 0.250,95%CI:0.152,0.349), *anxiety/depression*(*β* = 0.271, 95%CI:0.150, 0.392), and EQ-VAS scores(β = -3.027,95%CI:-3.641, -2.412). Regarding the relation between each individual type of MCI and HRQOL, The four complications all showed significant associations with the proportion of reported problems in certain dimensions. The pressure ulcer group showed significant association with all EQ-5D dimensions except *pain/discomfort*. DVT was found significantly associated with *mobility*, *usual activities* and *pain/discomfort*. Pneumonia was found to have significant association with all of the EQ-5D dimensions and EQ-VAS score except *usual activities* and *pain/discomfort*. The UTI group showed significant difference in *self-care* and EQ-VAS score when compared with patients without UTI.

**Table 3:** The adjusted effect of MCI on EQ-5D dimensions and EQ-VAS in relation to total and separated complications based on multilevel mixed-effects model (n = 18391).

Complications	EQ-5D dimensions(β,95%CI)					EQ-VAS(β,95%CI)
	Mobility	self-care	usual activities	pain/discomfort	Anxiety/depression	
**At least one (no = 0,yes = 1)**	0.229(0.127,0.331) ***	0.250(0.146,0.354) ***	0.250(0.152,0.349) ***	0.103(-0.005,0.221)	0.271(0.150,0.392) ***	-3.027(-3.641,-2.412) ***
**Press ulcer** **(no = 0,yes = 1)**	0.687(0.491,0. 883) ***	0.626(0.429,0.822) ***	0.641(0.444,0.839) ***	0.166(-0.052,0.383)	0.473(0.237,0.708) ***	-3.083 (-4.313,-1.853) ***
**DVT** **(no = 0,yes = 1)**	0.298(0.056,0.541) *	0.063(-0.189,0.325)	0.331(- = 0.093,0.570) **	0.320(0.069,0.569) *	-0.033(-0.338,0.272)	0.042(-1.465,1.549)
**Pneumonia** **(no = 0,yes = 1)**	0.069(-0.055,0.192) ***	0.154(0.003,0.278) *	0.111 (-0.007,0.230)	0.055(-0.073,0.182)	0.303(0.162,0.438) ***	-3.198 (-3.931,-2.465) ***
**UTI** **(no = 0,yes = 1)**	0.242(-0.040,0.524)	0.348(-0.064,0.632) *	0.230(-0.047,0.508)	-0.114(-0.408,0.179)	0.177(-0.134,0.487)	-5.256(-6.971,-3.540) ***

Note: Controls included hospital characteristics (hospital type), specialty of unit (ward classification) and patient characteristics (age, gender, education, physical activity status at admission, bedridden time, length of stay, number of diagnosis when discharge, modified Braden scale score, age adjusted Charlson comorbidity index).

CI, confidence interval; MICs, major immobility complications; DVT, lower limb deep vein thrombosis; UTI, urinary tract infections.

* p<0.05.

** p<0.01.

***p<0.001.

## Discussion

Results of this study highlight the adverse impact of MCI on bedridden patients. The researchers found that patients with any one type of MCI during hospitalization reported having more problems in every dimension of the EQ-5D and had substantially lower EQ-VAS mean scores than those without MCI. Experiencing at least one type of MCI had a significant association with all dimensions of EQ-5D except *pain/discomfort*, and EQ-VAS after adjustment for confounding variables. In addition, each individual type of MCI had a specific pattern of effect in relation to the dimensions of the EQ-5D.

Among patients who did not have any MCI during hospitalization, the proportion of problems reported using the EQ-5D at 3 months were slightly higher compared to population norms for the general Chinese adult population of similar age and gender. The EQ-VAS score was higher than that of a national representative general population in China (VAS = ~80) [29, 30]. Hence, on average, bedridden patients who do not develop MCI can expect that at the 3 month limit, their HRQOL will improve to a level similar to that of the general population. In contrast, among patients who developed MCI, their reported problems using EQ-5D were higher than that reported for general Chinese population of similar age and gender [29]. This suggests that MCI during hospitalization will still have a persistent negative association with HRQOL even after discharge.

The current analysis reveals that a high proportion of the patients with at least one type of MCI tended to report more problems with *usual activities* (48.7%), *mobility* (41.53%) *and self-care* (41.45%). This situation may be due partly to the reality that most (67.59%) of the patients with at least one type of MCI were older than 55, a population that is more likely to experience activity problems and impaired ability to meet their daily needs completely. They may require more care from caregivers.

Although patients from this population reported the problems of *pain/discomfort* and *anxiety/depression* less frequently (*pain/discomfort* 27.09%; *anxiety/depression* 20.5%) than problems in other EQ-5D dimensions, these proportions are still higher in comparison with patients without MCI (*pain/discomfort* 18.82%; *anxiety/depression* 10.9%). This finding indicates that MCI could have physiological and psychological impact on patients. There is accumulating evidence to suggest that MCI could have physical, social, and psychological impact on patients [31–34]. A systematic review showed that patients with pressure ulcer commonly reported negative emotions such as frustration, anxiety, and depression [31]. These findings imply that professional staff need to pay more attention to the psychological impact when caring for the patients with MCI.

In the multilevel mix-effects model, the researchers found that the presence of MCI was associated with decreased level of HRQOL. Experiencing any type of MCI during hospitalization raises the likelihood that patients will have more problems with dimensions of EQ-5D (except for *pain/discomfort*) and lower EQ-VAS scores. These findings illustrate that MCI have a negative persistent correlation with HRQOL, even after patients have been discharged.

All the four complications were found to have unique effect on HRQOL. Pneumonia is the most prevalent complication in this study population. We found that pneumonia was associated with lower level of HRQOL in all five dimensions of EQ-5D and EQ-VAS except for *usual activities and pain/discomfort*. This result is similar with previous studies conducted in community-acquired pneumonia patients [14], which found that HRQOL was persistently lower in community-acquired pneumonia patients compared to non-diseased persons. Recent studies recognize long-term adverse outcomes associated with pneumonia, including mortality [35] and reduced HRQOL [36]. Peyrani and colleague argued that patients surviving an episode of community acquired pneumonia were still at increased risk of death long after hospital discharge [37]. Our data add to the literature by demonstrating the significant link between pneumonia and HRQOL after a 3-month period. Regarding pressure ulcer and UTI, the pressure ulcer group showed significant association with all EQ-5D dimensions except *pain/discomfort*, while the UTI group showed significant associations with *self-care* and EQ-VAS. These results agrees with previous investigations in pressure ulcer [38] and UTI [20], which demonstrated that the two complications have an independent correlation with HRQOL.

On the other hand, we also observed a significant association between DVT and decreased HRQOL in the mixed-effects models. Utne et al. found that patients with DVT scored significantly lower on all dimensions of EQ‑5D when compared with buddy controls and population norms. In addition, *pain/discomfort* was the most common complaint in the patient group[13]. The author argued that the results may be due to pain being a symptom of post-thrombotic syndrome (PTS). The increased risk of patients indicating problems in the dimensions of *mobility* and *usual activities* may also be explained by body movement restrictions due to symptoms of PTS[39]. In the current study, we did not measure PTS among patients diagnosed with DVT. Further study is needed to explore the effect of DVT on HRQOL among bedridden patients with taking PTS into consideration.

Overall, the current research have important implications for providing care for bedridden patients. The result emphasized the need to adopt optimal strategies to prevent MCI during hospitalization. Furthermore, patients who have experienced MCI should be actively screened for these problems at routine follow-ups and offered rehabilitation (eg, therapeutic exercise and psychological support).

### Strengths and limitations

The current study is the first study that provides detailed HRQOL of bedridden patient with and without MCI for 3- month period using the EQ-5D. The major strength of this study is the large population of consecutively included and prospectively followed patients. Another strength is that the data set was prospectively maintained, detailed, and thorough in accordance with a predefined study protocol. This may have lowered the risk of systematic error due to misclassification.

A number of limitations in this study warrant mentioning., First, potential confounding factors associated with HRQOL included demographic (age, gender), socioeconomic (residency, marital status, educational attainment, employment, income, housing, and health insurance), and health (chronic conditions) variables [40]. Other confounding variables may exist. Future studies need to include large-scale related factors. Second, this study may have been influenced by a healthy participant effect, as the study population consisted of subjects who were willing to participate in a follow-up study. They may have been healthier than non-responding patients. Indeed, one of the major arguments for patients refusing participation was self-perceived poor health, their reported problems attributable to MCI would most likely have been higher. As a result, the observed effect value attributable to MCI during hospitalization may have been underestimated. There is also the issue of the validity of DVT and pneumonia diagnoses, which are based on the attending physician’s reported diagnosis. Regarding DVT, although the incidence of DVT in the current study (1.67%) was similar to that found in another study conducted in China (1.7%–2%)[41], not all patients were regularly screened for DVT, so we cannot exclude the possibility that some patients with DVT were not identified. If that were the case, the misclassification of DVT diagnosis would be expected to reduce the effect of DVT on HRQOL. Regarding pneumonia, although doctors in the participating hospitals used the same procedures and criteria to diagnose pneumonia (based on symptoms, a clinical exam, and a chest X-ray) [42], we did not have information on X-ray findings to confirm the diagnoses. These limitations suggest that caution should be taken in drawing firm conclusions.

## Conclusions

In summary, the findings from the large sample size study about the association between MCI and HRQOL have important implications for the care of those with bedridden patients. In particular, MCI independently serve to decrease HRQOL after discharge. It is clear that additional attention must be paid to bedridden patients with MCI in order to ensure that they enjoy a quality of life comparable to that of those without these health problems. Improvements to both quality of life and disease recovery could be achieved by improving the prevention and management of MCI among bedridden patients.
